# *Drosophila *insulin‐like peptide *dilp1 *increases lifespan and glucagon‐like Akh expression epistatic to *dilp2*

**DOI:** 10.1111/acel.12863

**Published:** 2018-12-03

**Authors:** Stephanie Post, Sifang Liao, Rochele Yamamoto, Jan A. Veenstra, Dick R. Nässel, Marc Tatar

**Affiliations:** ^1^ Department of Molecular Biology, Cell Biology and Biochemistry Brown University Providence Rhode Island; ^2^ Department of Ecology and Evolutionary Biology Brown University Providence Rhode Island; ^3^ Department of Zoology Stockholm University Stockholm Sweden; ^4^ Institut de Neurosciences Cognitives et Intégratives d’Aquitaine (CNRS UMR5287) University of Bordeaux Pessac France

**Keywords:** aging, Akh, *dilp1*, *dilp2*, *Drosophila*, insulin, insulin/IGF signaling

## Abstract

Insulin/IGF signaling (IIS) regulates essential processes including development, metabolism, and aging. The *Drosophila *genome encodes eight insulin/IGF‐like peptide (*dilp*) paralogs, including tandem‐encoded *dilp1 *and *dilp2*. Many reports show that longevity is increased by manipulations that decrease DILP2 levels. It has been shown that *dilp1* is expressed primarily in pupal stages, but also during adult reproductive diapause. Here, we find that *dilp1* is also highly expressed in adult *dilp2* mutants under nondiapause conditions. The inverse expression of *dilp1* and *dilp2* suggests these genes interact to regulate aging. Here, we study *dilp1* and *dilp2* single and double mutants to describe epistatic and synergistic interactions affecting longevity, metabolism, and adipokinetic hormone (AKH), the functional homolog of glucagon. Mutants of *dilp2* extend lifespan and increase *Akh *mRNA and protein in a *dilp1*‐dependent manner. Loss of *dilp1* alone has no impact on these traits, whereas transgene expression of *dilp1* increases lifespan in *dilp1* − *dilp2* double mutants. On the other hand, *dilp1* and *dilp2* redundantly or synergistically interact to control circulating sugar, starvation resistance, and compensatory *dilp5* expression. These interactions do not correlate with patterns for how *dilp1* and *dilp2* affect longevity and AKH. Thus, repression or loss of *dilp2* slows aging because its depletion induces *dilp1*, which acts as a pro‐longevity factor. Likewise, *dilp2* regulates *Akh* through epistatic interaction with *dilp1*. *Akh* and glycogen affect aging in *Caenorhabditis elegans *and *Drosophila*. Our data suggest that *dilp2* modulates lifespan in part by regulating *Akh*, and by repressing *dilp1*, which acts as a pro‐longevity insulin‐like peptide.

## 1 INTRODUCTION

Insulin/IGF signaling (IIS) is a fundamental pathway that regulates aging, development, metabolism, growth, and reproduction. The *Drosophila melanogaster *genome encodes several insulin‐like peptide genes (*dilps*) that signal through a single insulin‐like receptor (*InR*) (Brogiolo et al., 2001; Colombani, Andersen, & Leopold, 2012; Garofalo, 2002; Grönke, Clarke, Broughton, Andrews, & Partridge, 2010). Among their physiological functions, *dilps* regulate aging: Mutation of *dilp2* alone is sufficient to extend lifespan, whereas loss of other *dilps* does not (Grönke et al., 2010). How reduction of one specific *dilp *modulates aging is not understood. Here, we demonstrate that *dilp1* is upregulated in the absence of *dilp2*, that *dilp1* expression is required for loss of *dilp2* to slow aging, and that exogenous expression of *dilp1* in a *dilp1‐2* mutant is sufficient to extend lifespan.

The *dilp1 *gene is encoded approximately 1.2 kb upstream of *dilp2*, potentially as a result of a tandem duplication event (Tatar, Bartke, & Antebi, 2003). These paralogs are expressed in different developmental and life history stages. *Dilp2* is initially expressed in embryos and then throughout larval instar stages (Brogiolo et al., 2001; Slaidina, Delanoue, Gronke, Partridge, & Leopold, 2009). Pupae show decreased expression of *dilp2*, but the ligand is again highly expressed in adults. In contrast, during normal development, *dilp1* is only expressed in the pupal stage (Slaidina et al., 2009). While their timing is distinct, *dilp1* and *dilp2* are both expressed in median neurosecretory cells of the *Drosophila* brain, the insulin‐producing cells (IPCs) analogous to mammalian pancreatic β cells (Brogiolo et al., 2001; Liu, Liao, Veenstra, & Nässel, 2016; Rulifson, Kim, & Nusse, 2002).

The function of *dilps* in aging has been best studied for *dilp2*, *dilp3*, *dilp5,* and *dilp6 *(Bai, Kang, & Tatar, 2012; Broughton et al., 2010; Grönke et al., 2010). Lifespan is extended by mutation of *dilp2* alone, or *dilp2*, *dilp3,* and *dilp5* together: The normal function of *dilp2* appears to promote processes permissive to aging. On the other hand, induction of *dilp6* in fat body promotes longevity, perhaps because this decreases DILP2 peptide secreted from the IPCs (Bai et al., 2012). Similarly, increased *FOXO* expression in head fat body and increased JNK activity in IPCs extend lifespan, perhaps again because these manipulations decrease *dilp2* expression in the IPCs (Hwangbo, Gershman, Tu, Palmer, & Tatar, 2004; Wang, Bohmann, & Jasper, 2005). Across these studies, there has been no attention to *dilp1*. Yet notably, in contrast to normal laboratory conditions, DILP1 is produced in adult IPCs during reproductive diapause (Liu et al., 2016), which is a quiescent phase strongly associated with negligible aging (Tatar & Yin, 2001). The positive association between *dilp1* and diapause survival suggests this enigmatic insulin hormone may possess unusual functions in the control of aging.

Understanding how insulin peptides of *Drosophila* regulate aging is complicated by the fact that genetic or RNAi reduction of any one *dilp* gene induces compensatory expression in other *dilp* genes. For instance, a *dilp2* mutant increases expression of *dilp3* and *dilp5* (Grönke et al., 2010). Complex compensation and interaction is also known for *Caenorhabditis elegans* insulin‐like gene paralogs (Fernandes de Abreu et al., 2014). For instance, *ins‐6* is upregulated in an *ins‐23* mutant, and these paralogs appear to interact to regulate lifespan (Fernandes de Abreu et al., 2014). Notably, *C. elegans ins‐18* and *ins‐23* are proposed to function as insulin‐like receptor antagonists to regulate Dauer formation and favor longevity (Matsunaga, Matsukawa, Iwasaki, Nagata, & Kawano, 2018). To date, aside from the inverse regulation of aging by *dilp6* and *dilp2 *(Bai et al., 2012), functional interactions among *Drosophila* insulin paralogs have not been described.

Here, we study the relationship between *dilp1* and *dilp2*. We find that *dilp1* is strongly upregulated in *dilp2* mutants, consistent with *dilp1* serving a role in diapause conditions where it might regulate metabolism and slow aging. To test this model, we generated a *dilp1‐2* double mutant to complement revised *dilp1* and *dilp2* single mutants (Grönke et al., 2010). As previously reported, *dilp2* mutants are long‐lived. We now see that *dilp1* mutants have wild‐type longevity as do *dilp1* − *dilp2* double mutants; thus, loss of *dilp1* fully rescues the extended longevity of *dilp2*. We find that *dilp1* is also genetically downstream of *dilp2* in the control of *Drosophila* adipokinetic hormone (AKH), the functional homolog of mammalian glucagon. We confirmed the positive role of *dilp1* upon longevity and AKH by transgene *dilp1* expression in a *dilp1* − *dilp2* double mutant. In contrast to longevity and AKH, *dilp1* and *dilp2* do not epistatically control other tested physiological traits (e.g., hemolymph glucose or trehalose, starvation resistance, and glycogen), suggesting these phenotypes are not regulated through the same mechanisms by which these insulin‐like peptides interact to modulate aging. Our data together reveal a novel pathway by which a unique insulin‐like ligand, DILP1, positively regulates longevity.

## 2 RESULTS

**Figure 1:**
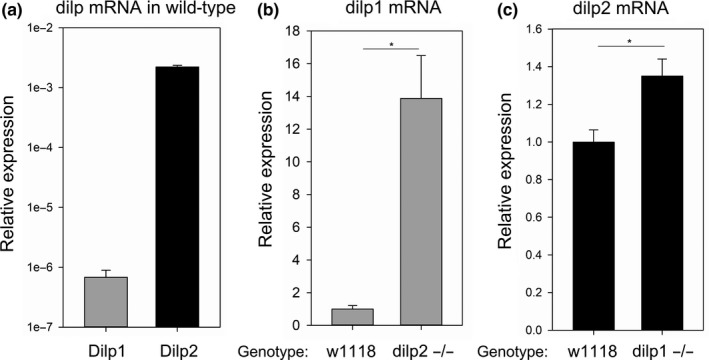
*Dilp1* mRNA is induced by depletion of *dilp2. *RNA from 7‐ to 10‐day‐old female adult flies was assayed by q‐RT–PCR. *n* = 6 per genotype. (a) *dilp1* mRNA expression is 100‐fold lower than *dilp2* expression in wild‐type flies. (b) *dilp1* mRNA expression increases 14‐fold in *dilp2* mutant flies compared to wild‐type flies, *t *test *p* < 0.001. (c) *dilp2* mRNA expression increases approximately 30% in *dilp1* mutant flies compared to wild‐type, *t *test *p* = 0.005

Studies on the control of aging by IIS in *Drosophila* have measured *dilp2*, *dilp3,* and *dilp5* mRNA or protein (Alic, Hoddinott, Vinti, & Partridge, 2011; Broughton et al., 2010; Hwangbo et al., 2004). While *dilp1* of the adult IPC is not observed in nondiapause conditions, we sought to characterize its expression in *dilp* mutants known to extend lifespan. In wild‐type adult females, *dilp1* mRNA is considerably lower than that of *dilp2* (Figure 1a). Strikingly, *dilp1* mRNA is elevated about 14‐fold in *dilp2* mutants relative to its expression in wild‐type (Figure 1b), while there is little compensatory expression of *dilp2* in *dilp1* mutants (Figure 1c). *Dilp2* appears to repress *dilp1*, and here, we test the proposition that *dilp1* may function in the absence of *dilp2* to regulate metabolism and aging.

### 2.1 Epistasis analysis of lifespan

Adult *dilp2* mutants have elevated blood sugar and extended lifespan (Grönke et al., 2010). To test whether these phenotypes require the expression of *dilp1*, we generated a *dilp1* − *dilp2* null double mutant by homologous recombination (HR), in parallel with matching *dilp1* and *dilp2* null single mutant knockouts (Supporting Information Figure S1). Previous studies evaluated *dilp* HR null mutants retaining the *white* marker gene, but we found these lines to disrupt gene expression of the nearby gene *Zasp67*. Importantly, *Zasp67* expression is not disrupted in our *white* marker excised lines (Supporting Information Figure S1). Similarly, *dilp2 *mutants of Grönke et al. (2010) that retain the *white* marker have increased expression of another gene near the *dilp *locus, *CG32052, *while this misexpression is absent from our *dilp2* mutants where the *white* marker is excised. Thus, using mutant lines without the *white* marker, if the functions of *dilp1* are redundant to those of *dilp2*, we expect the *dilp1* − *dilp2* double mutants to have greater longevity and higher blood sugar than either single mutant. Alternatively, if the functions of *dilp1* are downstream of *dilp2*, we expect the *dilp1* − *dilp2* double mutants to have wild‐type lifespan and metabolism.

**Figure 2:**
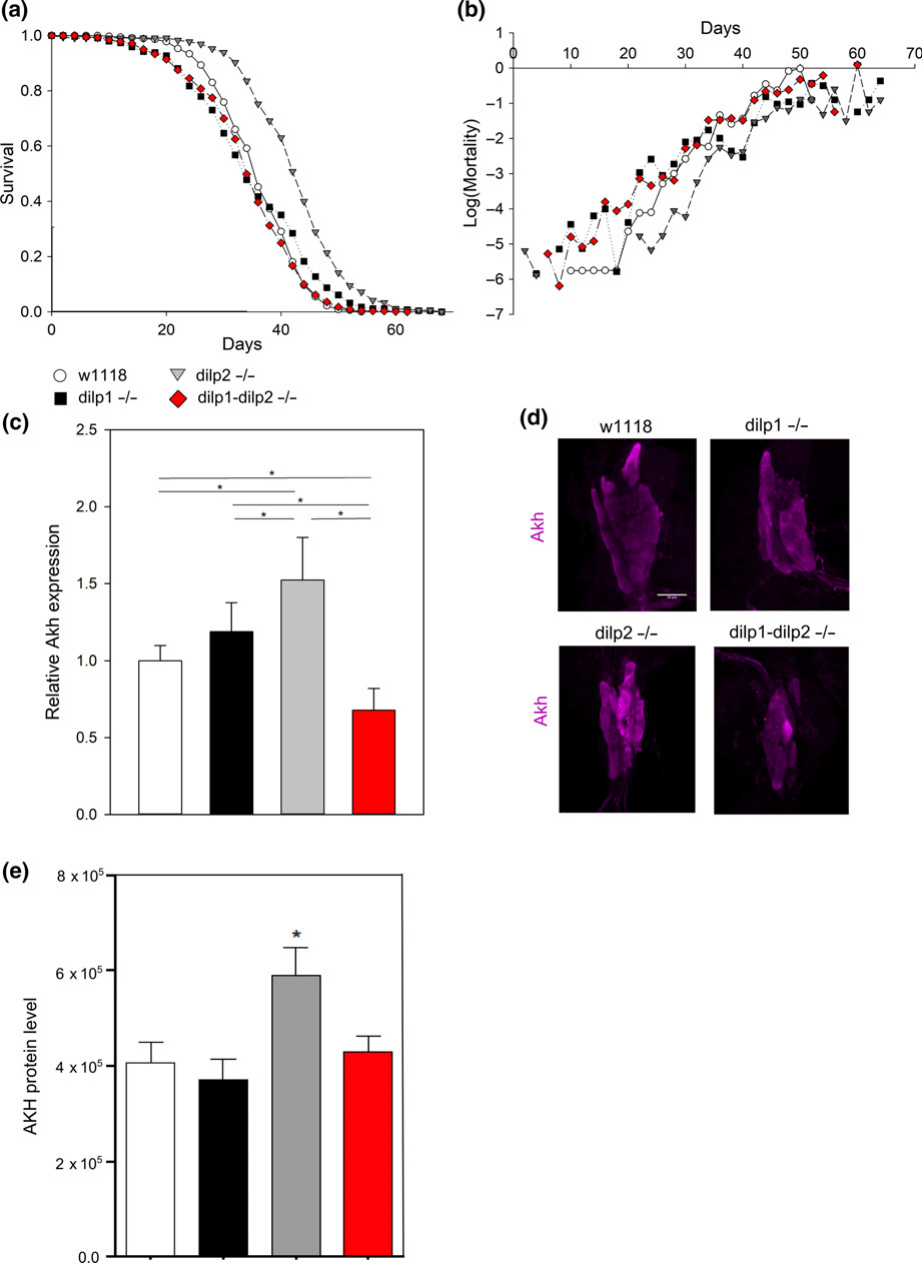
*dilp1* mutation suppresses aging and *Akh* phenotypes of mutant *dilp2*. (a) *dilp2* mutants but not double mutants are long‐lived, Cox hazard analysis *p* < 0.0001, *χ* ^2^ = 201, *n* = 341–365 per genotype. (b) *dilp2 *mutants but not double mutants have decreased mortality. (c) *dilp2* mutants but not double mutants have increased *Akh* mRNA expression, female adults at 7‐ to 10‐day‐old, *n* = 9 per genotype. Two‐way ANOVA *dilp1* *p ≤ *0.001, *dilp2 p* = 0.921, *dilp1 *× *dilp2* interaction *p* < 0.001. (D) *dilp2* mutants but not *dilp1* or double mutants have increased AKH immune‐labeling in corpora cardiaca from 6‐ to 7‐day‐old female flies, representative images. (E) Quantification of AKH immune‐labeling, *n* = 9–14 samples from three replicates, ANOVA *p < 0.05

The marker‐free null allele of *dilp2* increases lifespan by 20%–30% (Figure 2a, Supporting Information Figure S2d), confirming previous observations (Grönke et al., 2010). Null mutation of *dilp1* has no effect on adult survival, again as previously reported (Grönke et al., 2010). Remarkably, survival of the *dilp1* − *dilp2* double null mutant is indistinguishable from wild‐type or the *dilp1* mutant (Figure 2a,b), revealing a classic epistatic interaction between *dilp1* and *dilp2* in the control of longevity.

### 2.2 Epistasis analysis of adipokinetic hormone

*Dilp1 *is only normally expressed in adults during reproductive diapause, a slow‐aging stage associated with many metabolic changes, including elevated adipokinetic hormone (AKH), the functional homolog of mammalian glucagon (Kubrak, Kucerova, Theopold, & Nassel, 2014; Kucerova et al., 2016; Y. Liu et al., 2016). Accordingly, we studied how *dilp1* and *dilp2* affect AKH through genetic analysis of single and double mutants. *Akh* mRNA is increased in *dilp2* mutants, is similar to wild‐type in *dilp1* mutants, and is restored to wild‐type levels in the *dilp1* − *dilp2 *double mutant (Figure 2c). We likewise examined AKH immunostaining in the adult corpora cardiaca (CC). AKH peptide in the CC is increased in *dilp2* mutants and is similar to wild‐type in *dilp1* and in *dilp1* − *dilp2* double mutants (Figure 2d,e). These data suggest that *dilp1* is epistatically downstream of *dilp2* such that *dilp1* expression is required for *dilp2* to modulate lifespan and AKH.

### 2.3 Epistasis analysis of developmental and metabolic traits

**Figure 3:**
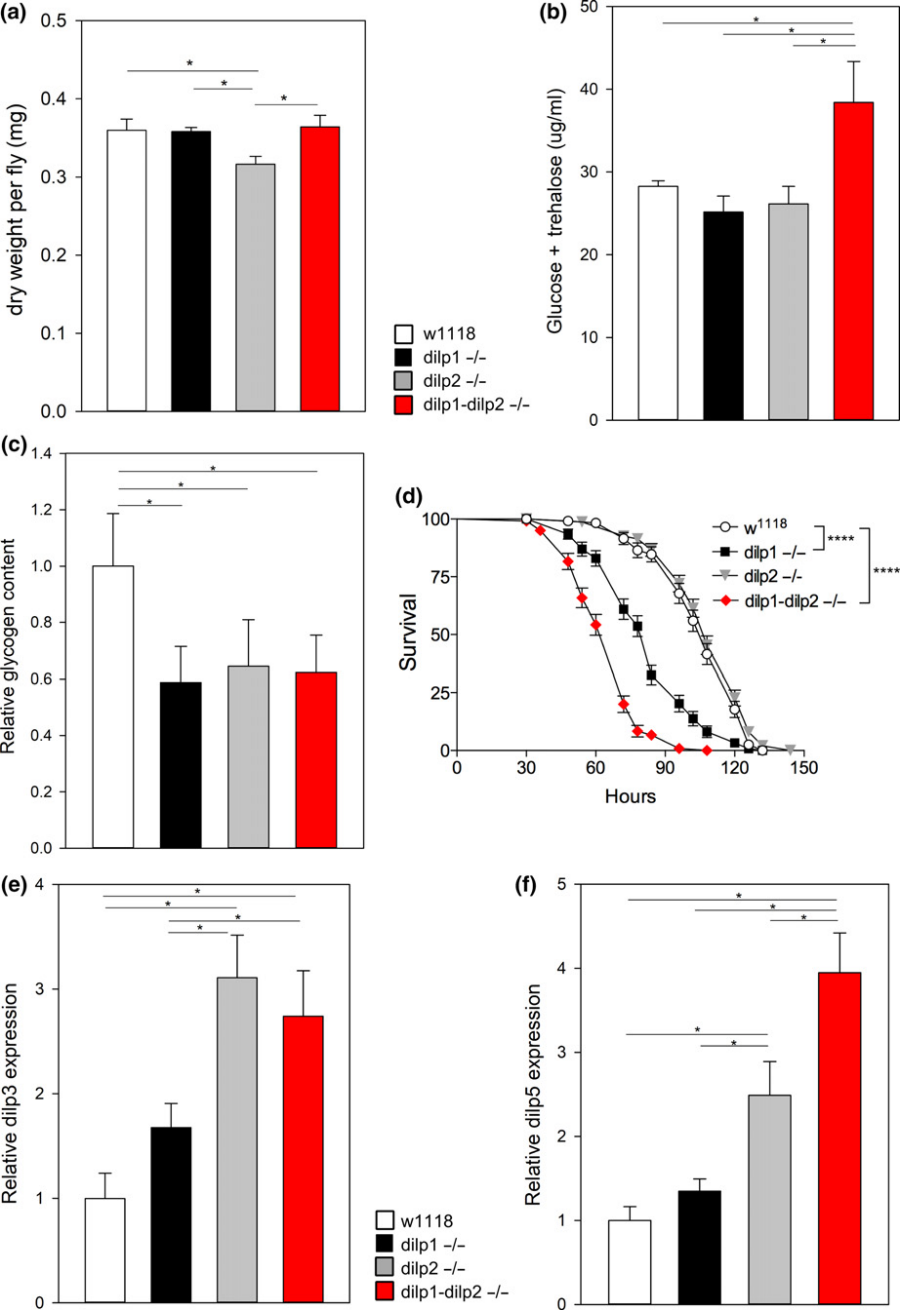
*dilp1* and *dilp2* interact to regulate metabolism, physiology, and compensatory *dilp* expression. Female flies aged 7–10 days old, 22–44 flies per replicate sample. Figure 3a–c,e,f show significance (*p < 0.05, **p < 0.01) from post hoc pairwise comparisons in two‐way ANOVA. (a) Adult mass reduced by *dilp2* mutant; *dilp1 *× *dilp2* interaction *p* = 0.03 (*n* = 5 samples per genotype). Hemolymph sugar (b) and glycogen (c) reduced by single dilp mutants, with significant *dilp1 *× *dilp2 *interaction, respectively, *p* < 0.001 and *p* = 0.005 (*n* = 5 samples per genotype). (d) Survival when fasted, each cohort with *n* = 118–150 flies, three replicate cohorts. Log‐rank tests relative to *w1118*, ****p < 0.0001. *dilp3* (e) and *dilp5* (f) mRNA are moderately induced by mutation of *dilp2* but not of *dilp1*, with significant *dilp1 *× *dilp2 *interaction, respectively, *p* = 0.02 and *p* = 0,02 (*n* = 3–6 replicate samples per genotype)

*Drosophila *insulins affect many traits including body weight and metabolism. Similar to the epistatic interactions observed for lifespan and AKH, body mass was decreased in *dilp2* mutants (as previously reported (Grönke et al., 2010)), but similar to wild‐type in *dilp1* mutants and in *dilp1* − *dilp2* double mutants (Figure 3a). In contrast, hemolymph (blood) glucose and trehalose concentrations in single mutants of *dilp1* and *dilp2* are similar to those seen in wild‐type, while the *dilp1* − *dilp2* double mutant has elevated hemolymph sugars (Figure 3b). For these traits, the insulin paralogs appear to have parallel, redundant functions. On the other hand, glycogen content is equally decreased by both single mutants and the double mutant, indicating that both *dilp1* and *dilp2* are required to maintain the pool of this energy storage molecule (Figure 3c).

Many longevity‐extending IIS manipulations increase resistance to fasting (Clancy et al., 2001; Grönke et al., 2010). In contrast to previous report (Grönke et al., 2010), survival during fasting for the long‐lived *dilp2* null genotype is similar to wild‐type, while fasted *dilp1* mutants and *dilp1* − *dilp2* double mutants are shorter lived (Figure 3d). These data suggest that *dilp1*, which is increased during nonfeeding developmental stages (Liu et al., 2016), may be required for starvation survival by inducing catabolism of nutrients.

*Dilp3* mRNA is increased in *dilp2* mutants and in *dilp1* − *dilp2* double mutants to a similar extent, but not significantly increased in *dilp1* mutants (Figure 3e). *Dilp5* mRNA is increased to a greater extent in *dilp1* − *dilp2* double mutants relative to its increase in either single mutant, representing synergistic genetic interaction between *dilp1* and *dilp2* (Figure 3f). We observed no induction or repression of mRNA for *dilp6* in single and double mutants of *dilp1* and *dilp2*, but slight increases in expression of *dilp7* and *dilp8* mRNA in *dilp1* and double mutants (Supporting Information Figure S2a–c).

Fecundity is not significantly different among wild‐type, single and double *dilp1* and *dilp2* mutants for adult females at one or three weeks old, although at two weeks old, *dilp2* mutants lay slightly more eggs per day than other genotypes (Supporting Information Figure S2e). Finally, egg‐to‐pupa viability was 50% less in *dilp1* − *dilp2* double mutants relative to wild‐type and to single mutants, suggesting that these insulin loci have redundant functions in larval survival (Supporting Information Figure S2f).

In sum, lifespan, body size, and AKH are mediated by genetic epistasis between *dilp1* and *dilp2*, where *dilp1* is inferred to function downstream of *dilp2*. Other measured phenotypes are jointly or independently regulated by *dilp1* and *dilp2*, in some cases by redundant functions of the ligands and in other cases through synergistic interactions.

### 2.4 Epistatic analysis of insulin/IGF and juvenile hormone signaling

**Figure 4:**
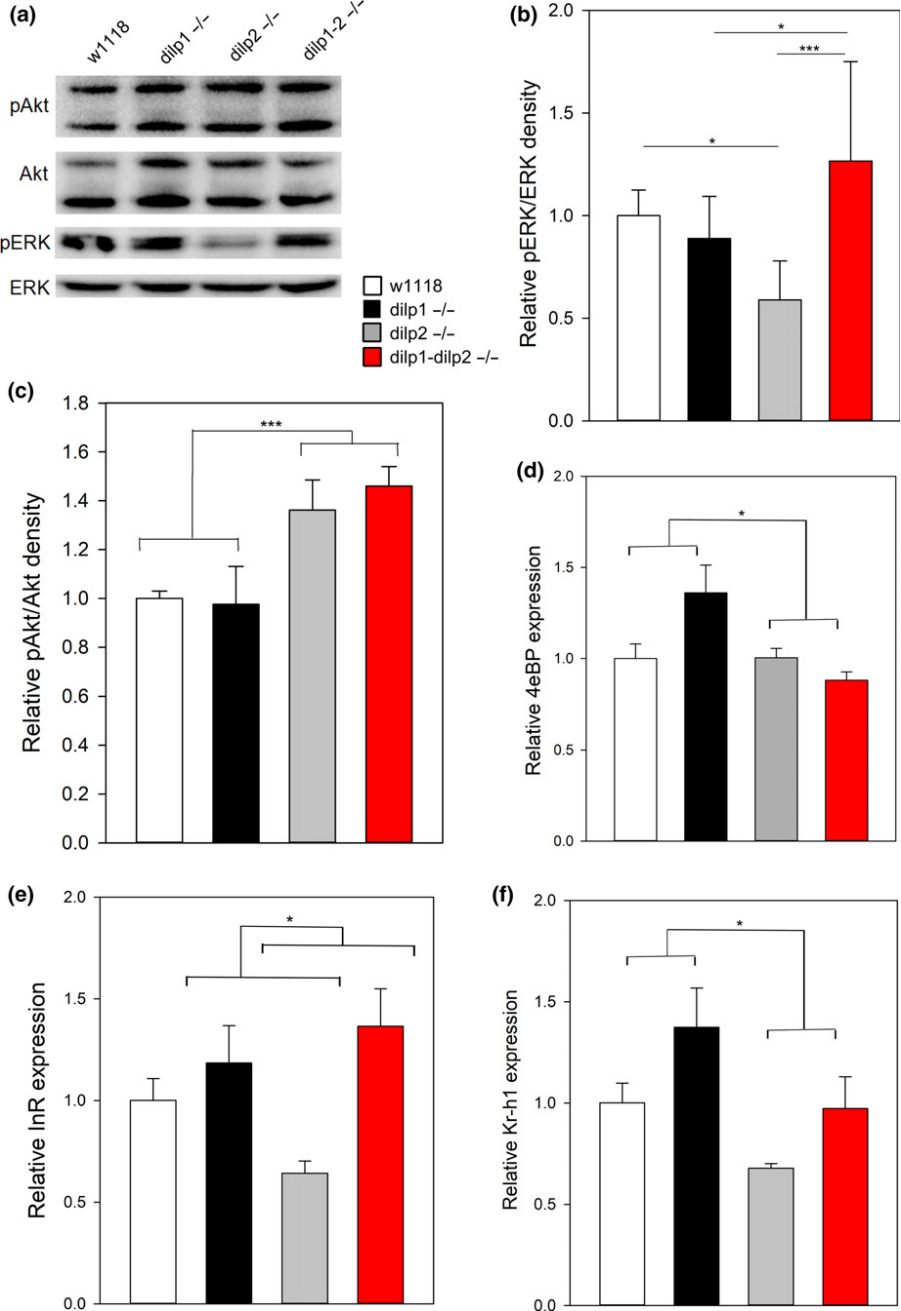
Components of insulin/IGF and JH signaling regulated by *dilp1* and *dilp2*. (a) pAkt in dissected thorax tissue is increased in *dilp2* and double mutants; pERK is decreased in *dilp2* mutants in a *dilp1*‐dependent manner, representative blot. Figures B‐F show significance (*p < 0.05, **p < 0.01) from post hoc pairwise comparisons in two‐way ANOVA. (b) Quantification of thorax pERK/ERK phospho‐westerns, *dilp1 *× *dilp2 *interaction *p* = 0.003, *n* = 6 per genotype. (c) Quantification of thorax pAkt/Akt phospho‐westerns, *dilp1 *× *dilp2 *interaction: not significant, *n* = 6 per genotype. (d) *4eBP* mRNA expression is not elevated in *dilp2* mutants but interacts with *dilp1*, *dilp1 *× *dilp2 *interaction *p* = 0.01, *n* = 7–9 per genotype. (e) *InR* mRNA expression is reduced in *dilp2* mutants relative to *dilp1*; *dilp2 *double mutant *p* < 0.05, *n* = 7–9 per genotype. (f) *Kr‐h1* mRNA expression is decreased by *dilp2* mutation, without significant *dilp1 *× *dilp2 *interaction, *n* = 8–9 per genotype

To understand how *dilp1* is required to extend longevity, we evaluated insulin/IGF signal (IIS) transduction and juvenile hormone (JH) signaling in single and double *dilp1* and *dilp2* mutants. Insulin ligands in *Drosophila* induce phosphorylation of Akt and ERK, which in turn regulate activity of transcription factors including FOXO. Here, we measured Akt and ERK phosphorylation from thorax tissue, which primarily consists of flight muscle (Figure 4a–c, Supporting Information Figure S3g). While loss of *dilp1* had no impact on Akt phosphorylation, loss of *dilp2* increased Akt phosphorylation in single and double mutants, suggesting that compensatory expression of other *dilps* (*dilp3* and *dilp5*) is sufficient to maintain and even elevate this branch of IIS in the absence of *dilp2*. In contrast, ERK phosphorylation in thorax is reduced in *dilp2* mutants, is unaffected in *dilp1* mutants, and is restored to wild‐type levels in the *dilp1* − *dilp2* double mutant. *Dilp1* and *dilp2* interact epistatically to control ERK phosphorylation. This pattern correlates with the epistatic interaction we observe for *dilp1* and *dilp2* in the control of longevity, and we note that ERK has been implicated in how IIS controls aging downstream of the insulin receptor substrate *chico* (Slack et al., 2015). In contrast, pAkt and pERK signaling measured from whole flies when fasted or fed was unaltered in *dilp1* and *dilp2* single and double mutants (Supporting Information Figure S3a–f). There is surprisingly little association among genotypes for longevity and systemically altered Akt or ERK activation.

Reduced IIS extends *Drosophila* lifespan in part through activating the FOXO transcription factor (Bai, Kang, Hernandez, & Tatar, 2013; Hwangbo et al., 2004; Giannakou, Goss, & Partridge, 2008; Min, Yamamoto, Buch, Pankratz, & Tatar, 2008) which subsequently induces target genes including *4eBP* and *InR*. Accordingly, we measured transcriptional targets of activated FOXO in *dilp1* and *dilp2* single and double mutants. Measured from whole animals, message from neither gene was elevated in the long‐lived *dilp2* single mutant, although some increase was seen in *dilp1* and *dilp1‐2 *mutants with normal lifespan (Figure 4d,e). Thus, in this mutant series, we find no association between longevity and elevated *4eBP* or *InR* expression, suggesting that activated FOXO may not be responsible for how reduced *dilp2* slows aging.

Juvenile hormone (JH) is an insect terpenoid hormone produced by the corpora allata that is documented to modulate how IIS impacts aging. The exceptional longevity of insulin receptor mutants is restored to wild‐type by treating adults with JH, while in flies with wild‐type IIS, eliminating adult JH production is sufficient to extend lifespan (Tatar et al., 2001; Yamamoto, Bai, Dolezal, Amdam, & Tatar, 2013). JH controls transcriptional programs by regulating expression and activation of the transcription factor Kruppel homolog 1 (Kr‐h1) (Liu et al., 2018; Minakuchi, Zhou, & Riddiford, 2008). Unlike for FOXO, *dilp1* and *dilp2* interact epistatically to control *Kr‐h1* mRNA, consistent with the epistatic interaction between *dilp1* and *dilp2* seen for longevity: *Kr‐h1* is reduced in *dilp2* mutants and restored to wild‐type expression in the *dilp1* − *dilp2* double mutant, while *dilp1* single mutants tend to have greater *Kr‐h1* mRNA than wild‐type (Figure 4f). *Dilp1* appears to normally repress JH activity, and we hypothesize this inhibition may be how longevity is extended by elevated *dilp1* when induced in *dilp2* mutants.

### 2.5 Overexpression of dilp1 rescues phenotypes in dilp1 − dilp2 double mutant

**Figure 5:**
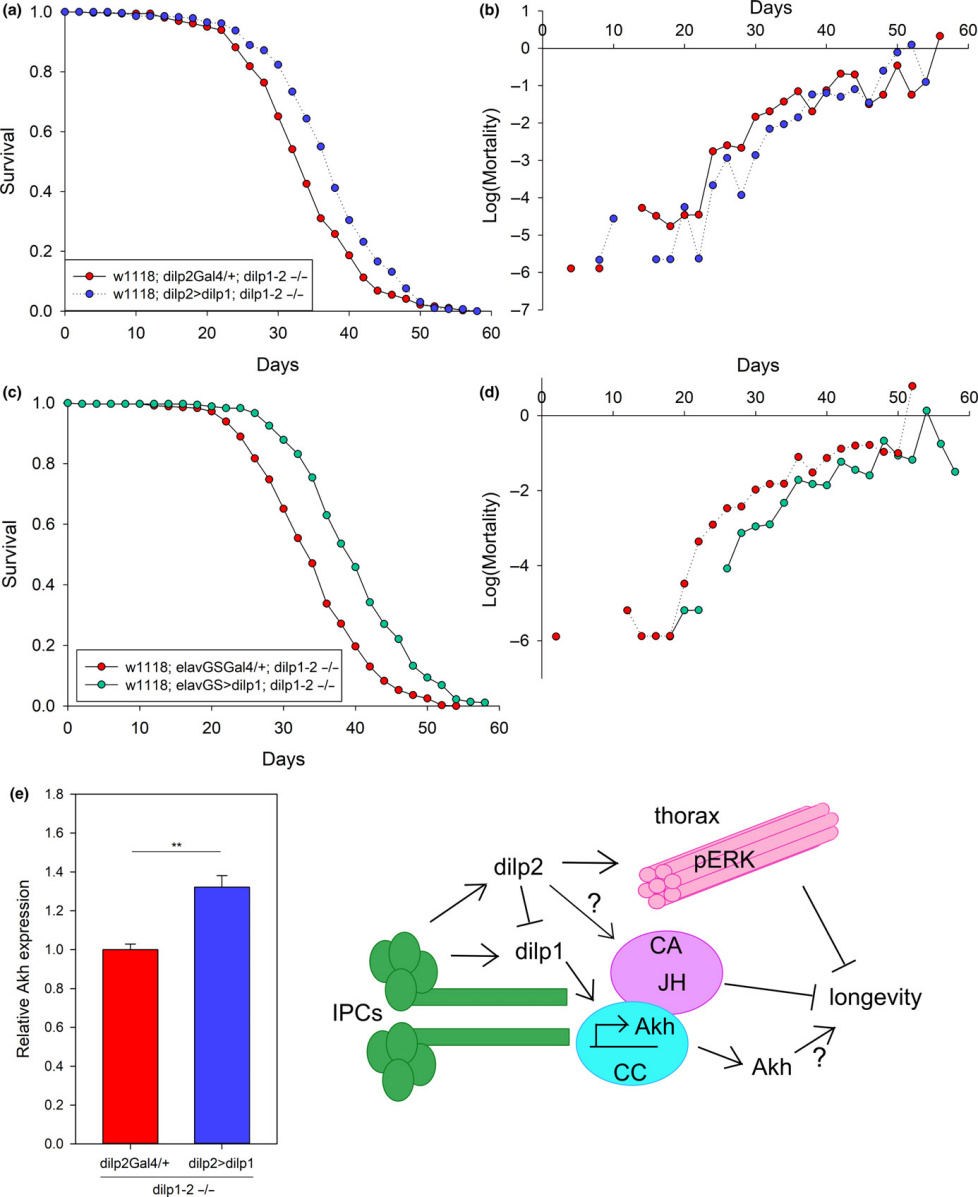
*Dilp1* expression in double mutants rescues longevity and AKH. (a) Lifespan is extended by *dilp2‐* GAL4>UAS‐*dilp1* overexpression rescue in the *dilp1* − *dilp2* double mutant background compared to *dilp2*‐GAL4/+ controls, Cox hazard analysis *p* < 0.0001, *χ* ^2^ = 25.8, *n* = 289–364 per genotype. (b) Mortality is decreased when *dilp2‐* GAL4>UAS‐*dilp1* is overexpressed in the *dilp1* − *dilp2* double mutants compared to *dilp2‐*GAL4/+ controls. (c) Lifespan is extended by *elav‐*GS>dilp1 overexpression in the *dilp1* − *dilp2* double mutant background treated with RU486 in adults relative to *elav*‐GS/+ controls, Cox hazard analysis *p* < 0.0001, *χ* ^2^ = 89.7, *n* = 361–377 per genotype. (d) Mortality is decreased by *elav*‐GS>UAS‐*dilp1* overexpression in the *dilp1* − *dilp2* double mutant background treated with RU486 in adults compared to *elav*‐GS/+ controls. (e) *Dilp2*>*dilp1* rescue in the *dilp1* − *dilp2* double mutant background increases *Akh* expression compared to *dilp2*‐Gal4/+ controls, *t *test *p* < 0.001, *n* = 5–6 per genotype. (f) Model for interaction between *dilp1* and *dilp2* in regulating lifespan. IPCs, insulin‐producing cells; CC, corpora cardiaca; CA, corpora allata; JH, juvenile hormone

We corroborate inferences on epistasis by exogenously expressing *dilp1. *We generated and validated a UAS‐*dilp1* stock capable of expressing this insulin protein using GAL4 drivers (Supporting Information Figure S4a). To verify whether loss of *dilp2* requires expression of *dilp1* to slow aging and increase *Akh* expression, we induced UAS‐*dilp1* in the double *dilp1* − *dilp2* null mutant background. Expressing exogenous *dilp1* in IPCs via *dilp2*‐GAL4 (Figure 5a,b, Supporting Information Figure S5a) or in all neurons with the RU486‐inducible GeneSwitch *elav*‐GSGal4 (Figure 5c,d) significantly extended lifespan by consistently decreasing age‐specific mortality. In one qualification, we note that *elav*‐GSGal4>*dilp1* flies not treated with RU486 (RU control cohort) nonetheless presented somewhat elevated *dilp1* expression and extended longevity relative to *elav*‐GSGal4/+ (genetic control cohort). Yet as required, survival of genetic controls was unaltered by RU486 treatment (Supporting Information Figure S5b). Likewise, *Akh* mRNA was elevated by *dilp1* transgene expression when driven by *dilp2*‐GAL4 in the double mutant background (Figure 5e). Confirmative outcomes were also seen when UAS‐*dilp1* was expressed in otherwise *dilp* wild‐type backgrounds: *dilp1* transgene expression in IPCs with *dilp2*‐GAL4 extended lifespan (Supporting Information Figure S6a,b).

DILP1 functions as a pro‐longevity factor, and it is necessary and sufficient for mutants of *dilp2 *to extend longevity. This result could be explained if DILP1 peptide inhibits insulin receptor tyrosine kinase activity. DILP1 might then slow aging by decreasing insulin/IGF signaling, indicated by reduced pAkt and pErk, induced FOXO target genes, and small body size. However, our current observations are inconsistent with this hypothesis. In long‐lived *dilp1‐2* double mutants with the *dilp1* transgene expressed in IPCs, pAkt and pERK in peripheral tissues were similar to levels seen in wild‐type (Supporting Information Figure S5e–g); among FOXO targets, *4eBp* and *InR* mRNA were slightly increased (Supporting Information Figure S5c); body mass was similar to wild‐type (Supporting Information Figure S5d). Likewise, in the long‐lived *dilp* wild‐type background with *dilp1* transgene expressed in IPCs, pAKT and pERK were not significantly reduced, *4eBP* mRNA was constant and *InR* mRNA was slightly repressed (Supporting Information Figure S6c–e). Overall, these data are contrary to expectations if DILP1 acts as an insulin receptor antagonist. Finally, we find few compensatory impacts on the expression of other *dilp* loci when *dilp1* is expressed in either mutant or wild‐type backgrounds (Supporting Information Figures S5h, Figure S6f), suggesting that feedback to the other *dilp* paralogs is not involved in this lifespan extension.

## 3 DISCUSSION

Based on mutational analyses of the insulin receptor (*daf‐2*, *InR*) and its associated adaptor proteins and signaling elements, numerous studies in *C. elegans* and *Drosophila* established that decreased insulin/IGF signaling (IIS) extends lifespan (Clancy et al., 2001; Kenyon, Chang, Gensch, Rudner, & Tabtiang, 1993; Tatar et al., 2001). Studies on how reduced IIS in *Drosophila* systemically slows aging also reveal systems of feedback where repressed IIS in peripheral tissue decreases DILP2 production in brain insulin‐producing cells (IPC), which may then reinforce a stable state of longevity assurance (Bai et al., 2012; Hwangbo et al., 2004; Wang et al., 2005). Here, we find that expression of *dilp1* is required for loss of *dilp2* to extend longevity (Figure 5f). This novel observation contrasts with conventional interpretations where reduced insulin ligand is required to slow aging: *Elevated dilp1* is associated with longevity in *dilp2* mutants, and transgene expression of *dilp1 increases *longevity.

*Dilp1* and *dilp2* are encoded in tandem, likely having arisen from a duplication event (Tatar et al., 2003). Perhaps as a result, some aspects of *dilp1* and *dilp2* are regulated in common: Both are expressed in IPCs (Liu et al., 2016; Rulifson et al., 2002), are regulated by sNPF (Lee et al., 2008), and have strongly correlated responses to dietary composition (Post & Tatar, 2016). Nonetheless, the paralogs are differentially expressed throughout development (Brogiolo et al., 2001). While *dilp2* is expressed in larvae, *dilp1* expression is elevated in the pupal stage when *dilp2* expression is minimal (Slaidina et al., 2009). In reproductive adults, *dilp1* expression decreases substantially after eclosion and *dilp2* expression increases (Slaidina et al., 2009).

Furthermore, DILP1 production is associated with adult reproductive diapause (Liu et al., 2016). IIS regulates adult reproductive diapause in *Drosophila*, a somatic state that prolongs survival during inclement seasons (Tatar & Yin, 2001). DILP1 may stimulate these diapause pro‐longevity pathways, while expression in nondiapause adults is sufficient to extend survival even in optimal environments.

Our data suggest a hypothesis whereby *dilp1* extends longevity in part through induction of adipokinetic hormone (AKH), which is also increased during reproductive diapause (Kucerova et al., 2016) and acts as a functional homolog of mammalian glucagon (Bednarova, Kodrik, & Krishnan, 2013). Critically, AKH secretion has been shown to increase *Drosophila* lifespan and to induce triacylglycerides and free fatty acid catabolism (Waterson et al., 2014). Here, we note that *dilp1 *mutants were more sensitive to starvation than wild‐type and *dilp2* mutants, as might occur if DILP1 and AKH help mobilize nutrients during fasting and diapause (Liu et al., 2016). Mammalian insulin and glucagon inversely regulate glucose storage and glycogen breakdown, while insulin decreases glucagon mRNA expression (Petersen, Vatner, & Shulman, 2017). We propose that DILP2 in *Drosophila* indirectly regulates AKH by repressing *dilp1* expression, while DILP1 otherwise induces AKH (Figure 5f).

A further connection between *dilp1 *and diapause involves juvenile hormone (JH). In many insects, adult reproductive diapause and its accompanied longevity are maintained by the absence of JH (Tatar & Yin, 2001). Furthermore, ablation of JH‐producing cells in adult *Drosophila *is sufficient to extend lifespan, and JH is greatly reduced in long‐lived *Drosophila* insulin receptor mutants (Tatar et al., 2001; Yamamoto et al., 2013). In each case, exogenous treatment of long‐lived flies with a JH analog (methoprene) restores survival to the level of wild‐type or nondiapause controls. JH is a terpenoid hormone that interacts with a transcriptional complex consisting of Met (methoprene tolerant), Taimen, and Kruppel homolog 1 (Kr‐h1) (Jindra, Bellés, & Shinoda, 2015). As well, JH induces expression of *kr‐h1 *mRNA, and this serves as a reliable proxy for functionally active JH. Here, we find that *dilp2 *mutants have reduced *kr‐h1* mRNA, while the titer of this message is similar to that of wild‐type in *dilp1* − *dilp2* double mutants. DILP1 may normally repress JH activity, as would occur in diapause when DILP1 is highly expressed. Such JH repression may contribute to longevity assurance during diapause as well as in *dilp2* mutant flies maintained in laboratory conditions.

Does DILP1 act as an insulin receptor agonist or inhibitor? Inhibitory DILP1 could directly interact with the insulin receptor to suppress IIS, potentially even in the presence of other insulin peptides. Such action could induce programs for longevity assurance that are associated with activated FOXO. Alternatively, DILP1 may act as a typical insulin receptor agonist that induces autophosphorylation and represses FOXO. In this case, to extend lifespan, DILP1 should stimulate cellular responses distinct from those produced by other insulin peptides such as DILP2 or DILP5 (Post et al., 2018). Through a third potential mechanism, DILP1 may interact with binding proteins such as IMPL2 or dALS to indirectly inhibit IIS output (Alic et al., 2011; Okamoto et al., 2013). We anticipate resolving these distinctions in a future study using synthetic DILP1 applied to cells in culture.

A precedent exists from *C. elegans* where some insulin‐like peptides are thought to function as antagonists (Matsunaga et al., 2018; Pierce et al., 2001). In genetic analyses, *ins‐23* and *ins‐18* stimulate larval diapause and longevity (Matsunaga et al., 2018), while *ins‐1* promotes Dauer formation during development and longevity in adulthood (Pierce et al., 2001). Moreover, *C. elegans ins‐6* acts through DAF‐2 to suppress *ins‐7* expression in neuronal circuits to affect olfactory learning, where *ins‐7* expression inhibits DAF‐2 signaling. These studies propose that additional amino acid residues of specific insulin peptides contribute to their distinct functions, and notably, the B‐chain of DILP1 has an extended *N*‐terminus relative to other DILP sequences (Brogiolo et al., 2001).

While dFOXO and DAF‐16 are intimately associated with how reduced IIS regulates aging in *Drosophila* and *C. elegans *(Martins, Lithgow, & Link, 2016), in our current work, the behavior of FOXO does not correspond with how longevity is controlled epistatically by *dilp1* and *dilp2*. Mutation of *dilp2* did not impact FOXO activity, as measured by expression of target genes *InR* and *4eBP*, and interactions with *dilp1 *did not modify this result. Some precedence suggests only a limited role for *dfoxo* as the mediator of reduced IIS in aging, as *dfoxo* only partially rescues longevity benefits of *chico *mutants, revealing that IIS extends lifespan through some FOXO‐independent pathways (Yamamoto & Tatar, 2011). On the other hand, *dilp1* expression from a transgene in the *dilp1–2* double mutant background did induce FOXO targets. Differences among these results might arise if whole animal analysis of dFOXO targets obscures its role when IIS regulates aging through actions in specific tissues (Tain et al., 2017; Wolkow, Kimura, Lee, & Ruvkun, 2000). In this vein, we find that *dilp2* controls thorax ERK signaling but not AKT, suggesting that *dilp2* mutants may activate muscle‐specific ERK/MAPK anti‐aging programs.

*Dilp1 *and *dilp2 *redundantly regulate glycogen levels and blood sugar, while these *dilp* loci interact synergistically to modulate *dilp5* expression and starvation sensitivity. In contrast, *dilp1* and *dilp2* interact in a classic epistatic fashion to modulate longevity and AKH. Such distinct types of genetic interactions may reflect unique ways DILP1 and DILP2 stimulate different outcomes from their common tyrosine kinase insulin‐like receptor, along with outcomes based on cell‐specific responses. Understanding how and what is stimulated by DILP1 in the absence of *dilp2* will likely reveal critical outputs that specify longevity assurance.

## 4 EXPERIMENTAL PROCEDURES

### 4.1 Fly husbandry

Flies were reared and maintained at 25°C, 40% relative humidity, and 12‐hr light/dark. Adults were maintained upon agar‐based diet with cornmeal (0.8%), sugar (10%), and yeast (2.5%). Fly stocks from Bloomington Stock Center include *w^1118^*, *dilp1* (#30,880) and *w^1118^*, *dilp2* (#30,881) mutants. *dilp2*‐Gal4 stock was originally obtained from Ernst Hafen, and *elav*‐GSGal4 stock was obtained from Steven Helfand (Brown University). All stocks were backcrossed to *w^1118^* for at least five generations.

### 4.2 Homologous Recombination

Homologous recombination (HR) of *dilp1* and *dilp2* in tandem was conducted as previously performed (Grönke et al., 2010; Staber, Gell, Jepson, & Reenan, 2011). See Supplemental Methods in Supporting Information Data S1.

### 4.3 Production of UAS‐*dilp1*

See Supporting Information Data S1, for detailed cloning procedures. Embryos were injected with UAS‐*dilp1* by BestGene Inc. (thebestgene.com) yielding five independent transformants for *dilp1*. For this study, we selected one transformant for *dilp1* that produced the strongest DILP1 immunolabeling when testing various Gal4 lines in larval and adult flies (see Supporting Information Figure S3).

### 4.4 Lifespan assays

Two‐ to three‐day‐old female adult flies, reared in density‐controlled bottles and mated after eclosion, were collected with light CO_2_ anesthesia and pooled in 1 L demography cages at a density of 100–125 flies per cage. Three independent cages were used per genotype. Food vials were changed every day for the first three weeks and then every two days for the remainder of each experiment. Dead flies were removed and recorded every other day. Cox proportional hazard analysis was conducted in R using the “surv” package and “survdiff” function.

### 4.5 RNA purification and quantitative RT–PCR

Total RNA was extracted from 20 whole mated female flies (8–10 days old) in TRIzol (Invitrogen, Grand Island, NY, USA) and treated with Turbo DNase (Invitrogen). RNA was quantified with a NanoDrop ND‐1000 (Thermo Fisher Scientific Inc., Wilmington, DE, USA) and reverse‐transcribed with iScript cDNA synthesis (Bio‐Rad Laboratories, Inc., Hercules, CA, USA). Quantitative RT–PCR was conducted with SYBR Green PCR master mix (Applied Biosystems, Carlsbad, CA, USA) and measured on an ABI PRISM 7,300 Sequence Detection System (Applied Biosystems). mRNA abundance was calculated by comparative CT relative to ribosomal protein 49 (RP49). Primer sequences are listed in Supporting Information Table S1.

### 4.6 Body Mass

Two females and two males in each vial were allowed to lay eggs for 24–36 hr or until proper density was attained (about 60–80 eggs). Eclosed flies were mated for two days, and females were sorted to separate vials. Food was changed every other day, and at 8–10 days old, flies were counted, briefly anesthetized on CO_2_, and collected in a preweighed microcentrifuge tube. Tubes were weighed, and mass per fly was calculated.

### 4.7 Western Blots

Antibodies from Cell Signaling Technology: *Drosophila* phospho‐Akt Ser505 (#4054S), Pan‐Akt (#4691S), Pan‐phospho‐ERK (#4370S), Pan‐ERK (#9102S). See Supplemental Methods in Supporting Information Data S1.

### 4.8 Antisera and immunocytochemistry

Tissues from larvae or 7‐day‐old female adults were dissected in 0.1 M PBS, then fixed for 4 hr in ice‐cold 4% paraformaldehyde (PFA), and rinsed in PBS three times for 1 hr. Incubation with primary antiserum was performed for 48 hr at 4°C. After rinsing in PBS with 0.25% Triton X‐100 (PBS‐Tx) four times, tissues were incubated with secondary antibody for 48 hr at 4°C. After washing in PBS‐Tx, tissues were mounted in 80% glycerol with 0.1 M PBS. Primary antisera used were as follows: rabbit antisera to DILP1 C‐peptide (Liu et al., 2016) at a dilution of 1:10,000, rabbit antisera to DILP2 and DILP3 A‐chains (Veenstra, Agricola, & Sellami, 2008) at a dilution of 1:2,000, rabbit antisera to AKH was kindly donated by M. Brown (Athens, GA) used at 1:1,000, and rabbit anti‐GFP at 1:000 (Invitrogen, Carlsbad, CA). Secondary antisera used were as follows: goat anti‐rabbit Alexa 546 antiserum and goat anti‐rabbit Alexa 488 antiserum (Invitrogen, Carlsbad, CA) at 1:1,000.

### 4.9 Image analysis

Confocal images were captured with a Zeiss LSM 780 confocal microscope (Jena, Germany) using a 40× oil immersion objective. The projection of z‐stacks was processed using Fiji (https://imagej.nih.gov/ij/). The cell body outlines were extracted manually, and the staining intensity was determined using Fiji. The background intensity for all samples was recorded by randomly selecting three small regions near the cell body of interest. The final intensity value of the cell bodies was determined by subtracting the background intensity.

## CONFLICT OF INTEREST

The authors declare no conflict of interest.

## AUTHOR CONTRIBUTIONS

SP and MT designed experiments and interpreted results. SP executed experiments. SF and RY executed experiments and contributed to experimental design. DN contributed to experimental design and interpretation of results. JV contributed to developing reagents. SP and MT wrote the manuscript and all authors edited the manuscript.
