# Supplementary figures and images for: Drosophila insulin‐like peptide dilp1 increases lifespan and glucagon‐like Akh expression epistatic to dilp2
Source: Aging Cell. 2018 Dec 3;18(1):e12863. doi: 10.1111/acel.12863 (PMC6351851; doi:10.1111/acel.12863)

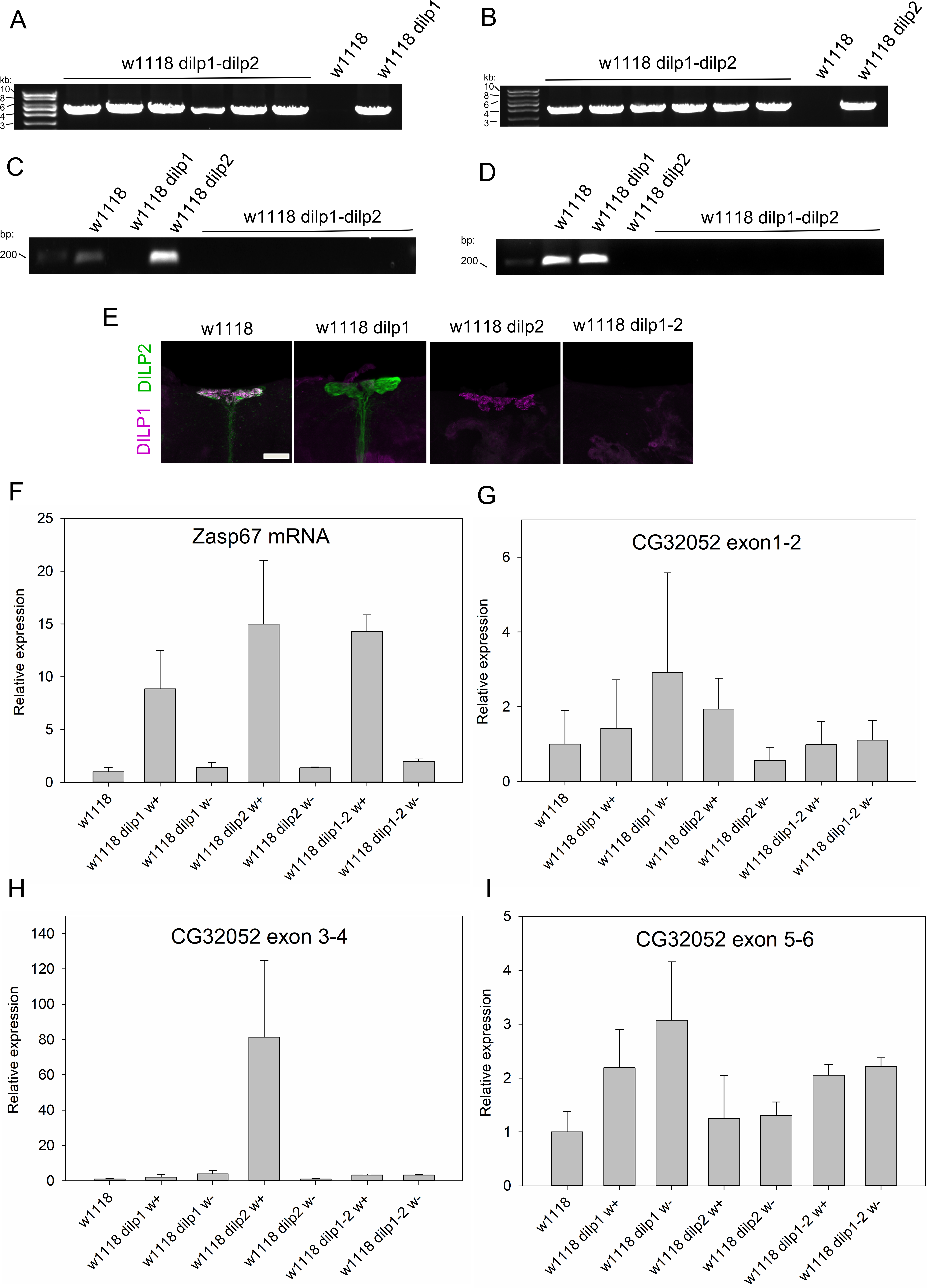

Supplement: Supplementary file 1 [file ACEL-18-e12863-s001.png]

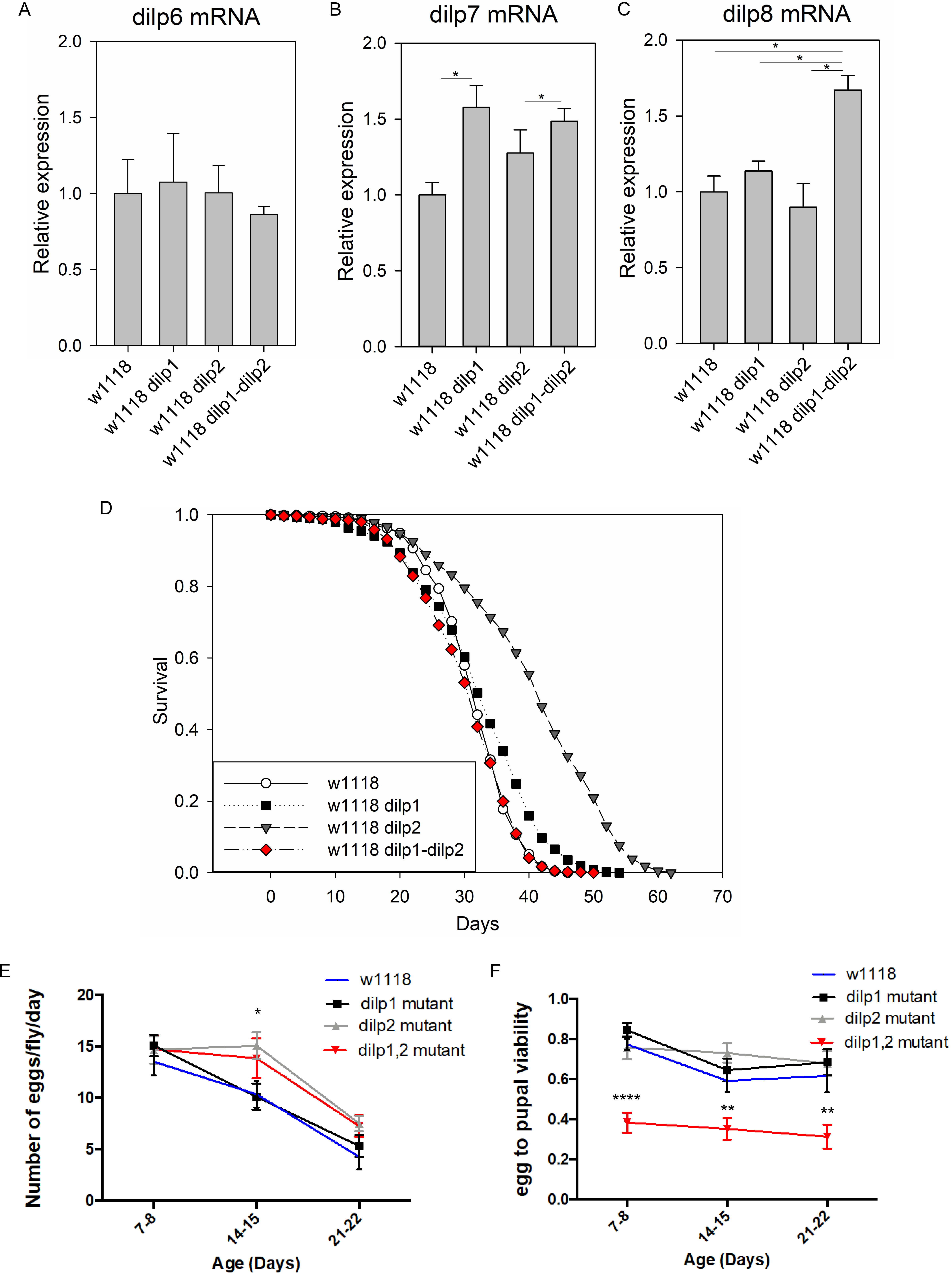

Supplement: Supplementary file 2 [file ACEL-18-e12863-s002.png]

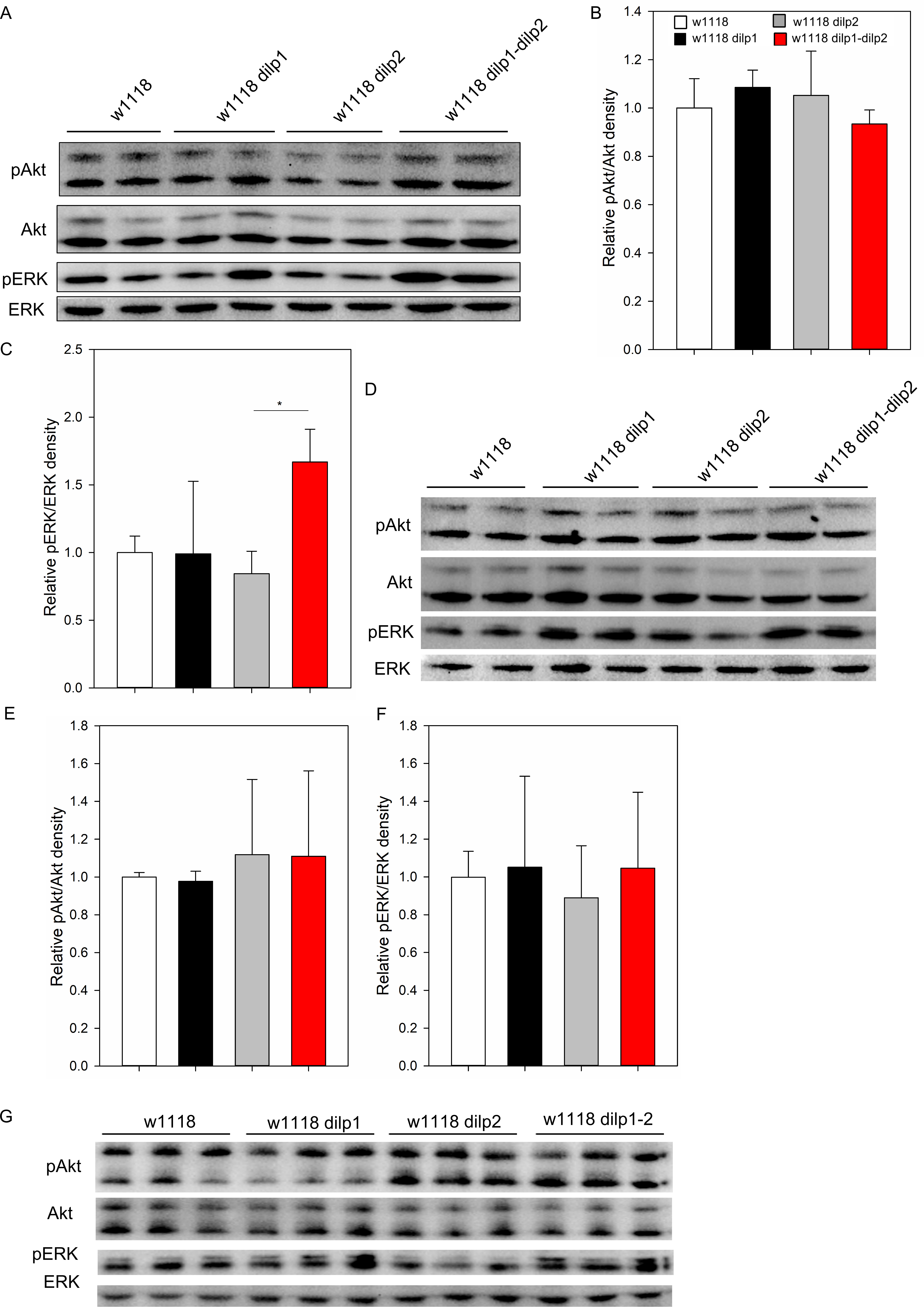

Supplement: Supplementary file 3 [file ACEL-18-e12863-s003.png]

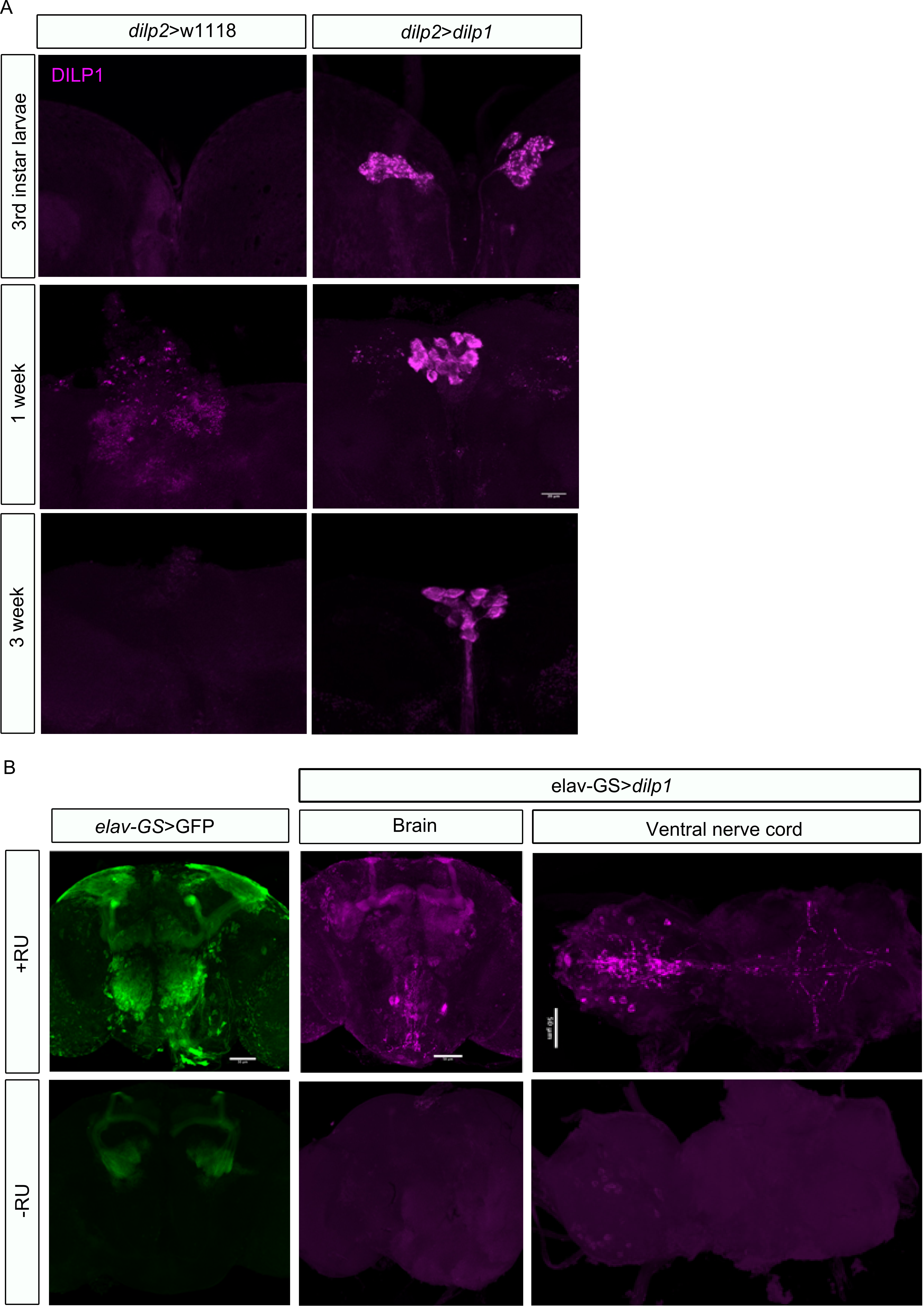

Supplement: Supplementary file 4 [file ACEL-18-e12863-s004.png]

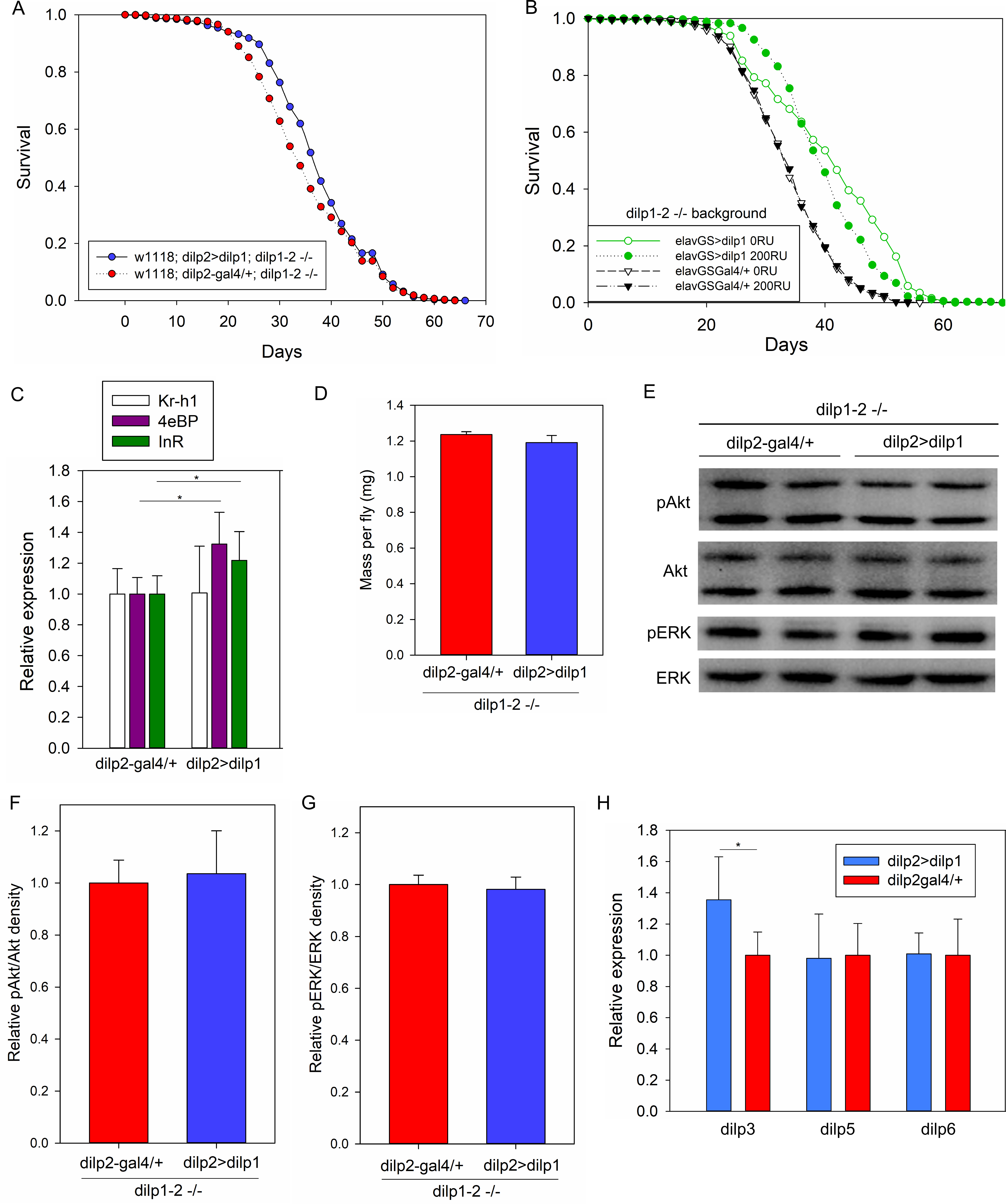

Supplement: Supplementary file 5 [file ACEL-18-e12863-s005.png]

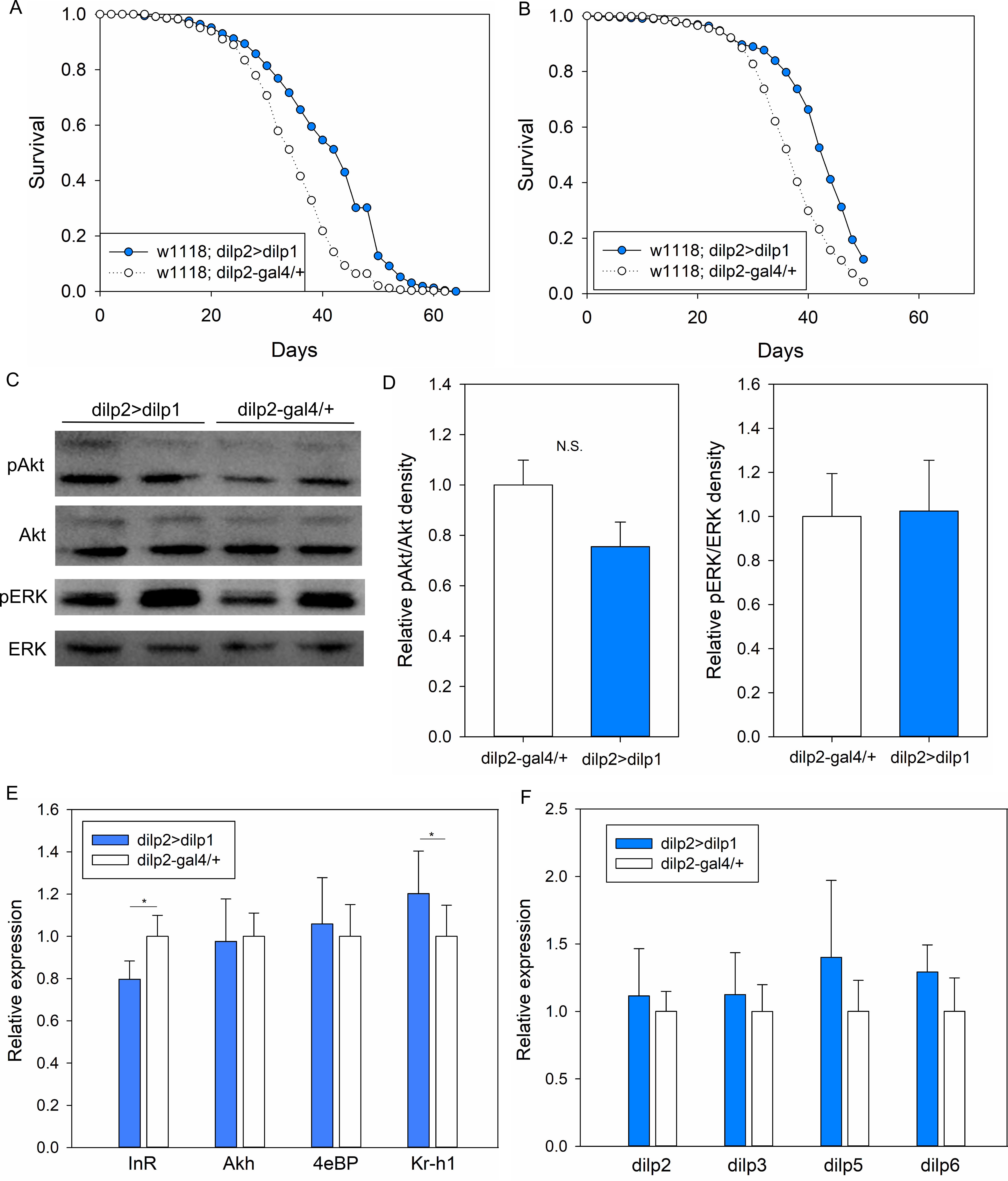

Supplement: Supplementary file 6 [file ACEL-18-e12863-s006.png]
