# Supplementary material for: Drosophila insulin‐like peptide dilp1 increases lifespan and glucagon‐like Akh expression epistatic to dilp2
Source: Aging Cell. 2018 Dec 3;18(1):e12863. doi: 10.1111/acel.12863 (PMC6351851; doi:10.1111/acel.12863)
Supplement: Supplementary file 7 [file ACEL-18-e12863-s007.docx]

Table S1. Primer Sequences

| ***Name*** | ***Sequence*** | ***Purpose*** | ***Reference*** |
| --- | --- | --- | --- |
| Dilp1F | GGGGCAGGATACTCTTTTAG | q-RT-PCR forward primer | Lee et al, 2009 |
| Dilp1R | TCGGTAGACAGTAGATGGCT | q-RT-PCR reverse primer | Lee et al, 2009 |
| Dilp2F | GTATGGTGTGCGAGGAGTAT | q-RT-PCR forward primer | Lee et al, 2009 |
| Dilp2R | TGAGTACACCCCCAAGATAG | q-RT-PCR reverse primer | Lee et al, 2009 |
| Dilp3F | AAGCTCTGTGTGTATGGCTT | q-RT-PCR forward primer | Lee et al, 2009 |
| Dilp3R | AGCACAATATCTCAGCACCT | q-RT-PCR reverse primer | Lee et al, 2009 |
| Dilp5F | AGTTCTCCTGTTCCTGATCC | q-RT-PCR forward primer | Lee et al, 2009 |
| Dilp5R | CAGTGAGTTCATGTGGTGAG | q-RT-PCR reverse primer | Lee et al, 2009 |
| Dilp6F | TGCTAGTCCTGGCCACCTTGTTCG | q-RT-PCR forward primer | Okamoto et al, 2009 |
| Dilp6R | GGAAATACATCGCCAAGGGCCACC | q-RT-PCR reverse primer | Okamoto et al, 2009 |
| Dilp7F | GAGCTGTACTCCTGTTCGTCCTGC | q-RT-PCR forward primer | Okamoto et al, 2009 |
| Dilp7R | TCCAAGCCTCATCATTGCCCGTCC | q-RT-PCR reverse primer | Okamoto et al, 2009 |
| Dilp8F | CTCAGCGAACTGGACATCTTT | q-RT-PCR forward primer | Post et al, 2016 |
| Dilp8R | CACTGGTTTAGACAGCAGTAGG | q-RT-PCR reverse primer | Post et al, 2016 |
| 4eBPF | CATAGCAGCCACACAAGCTC | q-RT-PCR forward primer | This study |
| 4eBPR | GGTGAAGCGGACATCTTAGC | q-RT-PCR reverse primer | This study |
| InRF | ACCTATTTAACCACAAGCGA | q-RT-PCR forward primer | Post et al, 2016 |
| InRR | CTCGATAGTTCCAAGATTGC | q-RT-PCR reverse primer | Post et al, 2016 |
| AkhF | GCGAAGTCCTCATTGCAGCCGT | q-RT-PCR forward primer | This study |
| AkhR | CCAATCCGGCGAGAAGGTCAATTGA | q-RT-PCR reverse primer | This study |
| Kr-h1F | TCA CAC ATC AAG AAG CCA ACT | q-RT-PCR forward primer | Kang et al, 2017 |
| Kr-h1R | GCTGGT TGG CGG AAT AGT AA | q-RT-PCR reverse primer | Kang et al, 2017 |
| RP49F | AAGAAGCGCACCAAGCACTTCATC | q-RT-PCR forward primer | Bai et al, 2013 |
| RP49R | TCTGTTGTCGATACCCTTGGGCTT | q-RT-PCR reverse primer | Bai et al, 2013 |
| Gr25SP | TAATTAATTGGTACCTACTTTATATTATTATTAATTTGGGACGTTACATTTATTATTATC | Amplifying homology arm 2 downstream of dilp2, forward | Gronke et al, 2010 |
| Gr26SP | CAAGGATCAGTATCATTTGGCATGCCCAGCGATCGGTTTGCCAAGAGCACGAGAAGTTCG | Amplifying homology arm 1 downstream of dilp2, reverse | Gronke et al, 2010 |
| Gr27SP | TAATTAATTCGTACGTGGAATACCTGAGATGGAGCCCTGGTTTCGGGGACACTCTAAGA | Amplifying homology arm 1 upstream of dilp1, forward | Gronke et al, 2010 |
| Gr28SP | TAATTAATTGGCGCGCCTCTTCTGCAGTCTAGCATTGCAGTGGCATAGCAAGCCCCACGG | Amplifying homology arm 1 upstream of dilp1, reverse | Gronke et al, 2010 |
| Gr54 | GAGAGGGAGAGTCACAAAACGAA | PCR verification of arm 2 forward primer | Gronke et al, 2010 |
| Gr55 | CGCTGCATGAATTAGCTTGGC | PCR verification of arm 1 reverse primer | Gronke et al, 2010 |
| Gr95 | GCCCTGCGTGGAGTAGAGTGTG | PCR verification of arm 2 reverse primer | Gronke et al, 2010 |
| Gr109 | CTGCAATTTCCTCCTCCACCAGC | PCR verification of arm 1 forward primer | Gronke et al, 2010 |
| Asc1 | GTATGCTATACGAAGTTATCTAGACTAGTCTAGGGCG | PCR verification of arm 1 in w- lines reverse primer | Staber et al, 2011 |
| Acc 1 | CATTATACGAAGTTATCTAGACTAGTCTAGGGTAC | PCR verification of arm 2 in w- lines forward primer | Staber et al, 2011 |
