# Supplementary material for: Drosophila insulin‐like peptide dilp1 increases lifespan and glucagon‐like Akh expression epistatic to dilp2
Source: Aging Cell. 2018 Dec 3;18(1):e12863. doi: 10.1111/acel.12863 (PMC6351851; doi:10.1111/acel.12863)
Supplement: Supplementary file 8 [file ACEL-18-e12863-s008.docx]

**Table S2. Leaky *dilp1* mRNA expression in *elav*-GS>*dilp1* controls untreated with RU in the *dilp1-2* double mutant background.** Expression was measured by quantative RT-PCR and represented relative to RP49 standard, n=3-6 per genotype. Wildtype w^1118^ and single *dilp2* mutants’ *dilp1* expression relative to RP49 is included for reference to the degree that *elav*-GS>*dilp1* induces overexpression and leaky expression.

| **Genotype** | **RU treatment** | **Relative *dilp1* mRNA abundance** | **Standard deviation** |
| --- | --- | --- | --- |
| w^1118^ ; UAS-*dilp1*/+ ; *elav*-GSGal4,*dilp1-2* | - | 0.0012 | +/- 5.8x10^-5^ |
| w^1118^ ; UAS-*dilp1*/+ ; *elav*-GSGal4,*dilp1-2* | + | 0.0971 | +/- 1.9x10^-2^ |
| w^1118^ ; +/+ ; *elav*-GSGal4,*dilp1-2* | - | None detected | N/A |
| w^1118^ ; +/+ ; *elav*-GSGal4,*dilp1-2* | + | None detected | N/A |
| w^1118^ | N/A | 3.37x10^-6^ | +/- 2.8x10^-6^ |
| w^1118^ ;; *dilp2* | N/A | 3.29x10^-5^ | +/- 2.6x10^-5^ |
