# Supplementary material for: Drosophila insulin‐like peptide dilp1 increases lifespan and glucagon‐like Akh expression epistatic to dilp2
Source: Aging Cell. 2018 Dec 3;18(1):e12863. doi: 10.1111/acel.12863 (PMC6351851; doi:10.1111/acel.12863)
Supplement: Supplementary file 9 [file ACEL-18-e12863-s009.docx]

**Supporting Information Data S1**

**Supplemental Methods**

***dilp1-dilp2* homologous recombination double mutants**

Homology regions ~4kb upstream of *dilp1* and ~4kb downstream of *dilp2* were amplified from wDah flies by PCR (NEB Phusion High Fidelity) with primers previously described in the process to produce *dilp1* and *dilp2* single mutants ([Grönke, Clarke, Broughton, Andrews, & Partridge, 2010](#_ENREF_1))(see Supplemental Table S1), and cloned into a pTOPO plasmid. Homology arms were subcloned into a pw25 vector. Vectors were sequenced to ensure patency and agreement with published coding regions. Vectors were transformed by P-element insertion and embryos injected at Genetic Services, Inc. (Cambridge, MA, USA). Ends-out HR crosses were conducted as previously performed (([Staber, Gell, Jepson, & Reenan, 2011](#_ENREF_2)). Following HR, *dilp1-dilp2* mutants were verified by genomic PCR and RNA quantitative RT-PCR, then backcrossed to *w^1118^* for at least five generations. Backcrossed mutants were crossed with flies expressing Cre recombinase (*yw*; CyO-Cre) to remove the mini-white marker flanked by loxP sites. Single mutants of *dilp1* and *dilp2* reported by Grönke et al. (2010) were obtained from the Bloomington Stock Center, backcrossed to *w^1118^* for at least five generations, then crossed to *yw*; CyO-Cre to remove the mini-white marker. Chromosomal exchange was used to replace the first and second chromosomes of the intermediary *yw* stock with chromosomes of the common *w^1118^* stock. These re-derived *dilp1* and *dilp2* solo mutants were verified by genomic PCR and RNA quantitative RT-PCR.

**UAS-*dilp1***

Expression via UAS-*dilp1* followed the protocol of Pfeiffer et al. (2012). From flies carrying the pJFRC29-10XUAS-IVSmyr::GFP-p19 transgene, a kind gift from Barry Pfeiffer, the 10XUAS region was amplified with the following primers: CCAGAATTCGGTCTCGGATCCGTCCTCAACGACAGGAGCAC and CGTAGGTACCGCCGCCTGAAGTAAAGGATA. The PCR product was digested with Kpn I and Eco RI and cloned into pUC19 digested with the same enzymes producing pUC19-10XUAS. The above mentioned flies were next used to amplify the terminator region using CGTAAGCTTGGTCTCGGATCCGAGGCCCTTTCGTCTTCAA and ATTACACACGGCATGGATGA. This PCR product was digested with Xba I and Hind III and cloned into pUC19-10XUAS digested with the same enzymes to create pUC19-10XUAS-Terminator. The *dilp-1* coding sequence, which lacks introns, was initially amplified from genomic DNA with CCCAGAGCATTCACTGCATA and GGACGATCTAGAGTGTGTGTTGGCCAAGTGTC. The resulting product was purified and re-amplified with GGACGATCTAGAGTGTGTGTTGGCCAAGTGTC and GATGGTACCAACTTAAAAAAAAAAATCAAAATGTTTAGCCAGCACAACGG. The latter PCR product was purified and cloned into pUC19-10XUAS-Terminator after digestion with Xba I and Kpn I to make UAS-*dilp1*-pUC19. The complete UAS-*dilp1* transgene was then removed from UAS-*dilp1*-pUC19 by digesting with Bsa I and ligating into the vector backbone of pUAST that had been digested with Bam HI. Sanger sequencing verified the transgene. UAS-*dilp1* transformant flies were backcrossed six times to *w1118* to generate *w1118*;UAS-*dilp1* lines and to recombine with *w1118;;dilp1-2* double mutants.

**Western Blots**

Mated female flies age 8-10 days were harvested in NP40 lysis buffer (Invitrogen) supplemented with 1mM PMSF, PhosSTOP phosphatase inhibitor cocktail (Roche #04906837001) and Protease Inhibitor Cocktail (Invitrogen). Samples were run on SDS-PAGE (Invitrogen NuPAGE). Gels were transferred to nitrocellulose membrane (Whatman) for one hour at 30V and washed in TBS-T for 10 min. Membranes were blocked with 5% BSA in TBS-T for one hour, and incubated with antibody 1:1000 in 5% BSA overnight at 4°C. Blots were washed 3 times for 5 min and incubated in HRP-conjugated anti-rabbit secondary antibody (Jackson Immunoresearch) 1:5000 in 1% BSA for one hour at room temperature. Blots were washed and incubated with ECL reagent (Perkin Elmer #NEL121001EA) for 5 minutes. Blots were imaged and analyzed by volume densitometry in ImageLab (BioRad). The density of phosphorylated protein was normalized with the total protein density as a loading control, and represented as a relative density of control set to one.

**Starvation resistance assays**

Flies were mated for two days and females were separated and maintained for 6-7 days on normal food. For fasting, the flies were kept in vials containing 5 ml of 0.5% agarose (Sigma-Aldrich). Dead flies were counted at least every 12 hours. No less than 118 flies from 3 replicates were used for the analysis.

**Glycogen quantification**

Mated female flies aged 8-10 days were flash frozen and homogenized in PBS. Samples were heat-treated at 70°C for 10 min and spun down at 14K rpm for 3 min at 4°C. Diluted amyloglucosidase enzyme (Sigma, #A1602) was added to the glycogen standard dilutions and half of the sample wells in a 96-well clear plate. PBS was added to the glucose standards and the other half of the sample wells. After 1 hour at 37°C, Glucose Hexokinase Reagent (Thermo Scientific) was added and the plate was incubated at room temperature for 15 min. The absorbance was read at 340nm on a SpectraMax M5 platereader using Softmax Pro software. The glycogen absorbance was quantified by subtracting the glucose absorbance from the total glycogen + glucose absorbance, and normalized to protein content by BCA assay.

**Hemolymph sugar quantification**

Mated female flies aged 8-10 days were decapitated to collect hemolymph in a microcentrifuge tube containing mesh. Flies were centrifuged for 4.5 min at 3K rpm and 4°C, and about 1ul hemolymph was transferred to 9ul PBS. Diluted hemolymph was heat-treated at 70°C for 5 min and transferred to tubes containing 200ul of Glucose Hexokinase Reagent (Thermo Scientific) with or without Porcine Kidney Trehalase (1:1000; Sigma-Aldrich). Reactions were incubated at 37°C for 16 hours and then transferred to wells of a clear microplate. The absorbance was read at 340nm using a SpectraMax M5 platereader and Softmax Pro software.

**Oviposition and egg to pupae viability**

Individual pairs of males and females were placed in vials containing fresh food. After 24 hours, the number of eggs laid was counted. Subsequently, the total number of pupae was counted and the viability of egg to pupae was determined as pupa number/egg number × 100%. 28-30 pairs of flies were analyzed at one, two and three weeks old.

**Supplemental Figure Legends**

**Fig S1. Validation of *dilp1-dilp2* mutants.** Genomic PCR verifies the homologous recombination event took place in the double mutant lines in the *dilp1* gene region (A) and *dilp2* gene region (B). RT-PCR of *dilp1* (C) and *dilp2* (D) mRNA verifies that the double mutants are null for both transcripts. Immunofluorescence of DILP1 and DILP2 protein (E) verifies that single mutants lack one protein and double mutants lack both proteins. Representative images shown. (F) *Zasp67* gene expression by qPCR is increased in *dilp* homologous recombination mutants retaining the mini-white marker gene, but is not increased in mutants with the marker excised by Cre recombination. (G-I) *CG32052*, a gene whose coding sequence overlaps the *dilp1-4* locus, has variable expression. Using qPCR primers targeting three different exon-exon junctions, expression does not vary significantly between wildtype, mini-white lines and excised lines between exon 1 and 2 (G) or exon 5 and 6 (I). *Dilp2* mutants with the mini-white marker have increased expression of *CG32052* at exon 3-4, but *dilp2* mutants with the marker excised have levels comparable with wildtype.

**Fig S2. Additional epistasis analyses between *dilp1* and *dilp2* single and double mutants.** (A) *Dilp6* mRNA levels do not significantly vary among single and double mutants. (B) *Dilp7* mRNA levelsincrease slightly in dilp1 and double mutants, two-way ANOVA dilp1 factor p=0.002, *p<0.05 post-hoc pairwise comparison, n=6-12 samples per genotype and ~20 flies per sample.. (C) *Dilp8* mRNA levels increase slightly in double mutants, two-way ANOVA dilp1xdilp2 interaction p=0.011, dilp1 factor p<0.001, n=6-12 samples per genotype and ~20 flies per sample. (D) Independent replicate life table study validates epistasis results of Figure 2A: the double mutant fully suppresses longevity conferred by *dilp2* mutation. (E) Fecundity (laid eggs/surviving female) does not differ among mutants and controls, except for *dilp2* mutants at 14-15 days, n=28-30 pairs of flies from three replicates, * p<0.05. (F) Larval viability is decreased in double mutants relative to wildtype, but does not vary significantly between single mutants and wildtype, n=16-30, n=28-30 pairs of flies from 3 replicates, two-way ANOVA ** p<0.01, **** p<0.0001.

**Fig S3. Additional IIS western blot analyses of *dilp1* and *dilp2* single and double mutants**

(A) Mutants starved for five hours have similar pAkt/Akt and pERK/ERK relative to wildtype, representative blot (prepared from whole animal); (B) quantified pAkt/Akt density does not vary among starved single and double mutants and wildtype, n=4 per genotype; (C) quantified starved pERK/ERK density does not vary among single mutants but is increased in double mutants (n=4 per genotype, ANOVA * p=0.012). (D) Fed mutants have similar pAkt/Akt and pERK/ERK relative to wildtype, representative blot (prepared from whole animal); (E) quantified pAkt/Akt density does not vary among fed single and double mutants and wildtype, n=3-4 per genotype; (F) quantified pERK/ERK density does not vary among fed single and double mutants and wildtype, n=3-4 per genotype. (G) Replicates of insulin/IGF signaling proteins from thorax, corroborating Fig 4A-C where pERK/ERK is decreased in *dilp2* mutants and pAkt/Akt is slightly increased in *dilp2* and double mutants.

**Fig S4. UAS-*dilp1* expression drives peptide production.**

(A) Overexpression of *dilp1* is induced by *dilp2*-Gal4 in IPCs. In control flies, DILP1 immunolabeling is undetectable in IPCs of 3rd instar larvae, very weak in 1-week-old adult and completely absent in 3-week-old flies. *dilp2*>*dilp1* flies show strong DILP1 signal in 3rd instar larvae, as well as in 1- and 3-week old adult flies. Scale bar 20 µm. (B) With GeneSwitch (GS), RU486 induces of GFP and *dilp1* during the adult stage (one week old). *Elav-GS*>GFP flies have strong GFP expression in many brain neurons with RU486 treatment, whereas only weak GFP is detected in the mushroom body and antennal lobe in the absence of RU486. With RU486 *Elav*-*GS*>*dilp1* drives strong DILP1 expression in many brain neurons as well as some cells of the ventral nerve cord (VNC). Weak or no DILP1 staining is seen in IPCs in the absence of RU486. Scale bar 50 µm.

**Fig S5. *dilp1* transgene does not repress insulin/IGF signaling or growth.**

(A) Independent, replicate lifetable study of *dilp1* transgene driven by *dilp2*-GAL4 in *dilp1; dilp2* null background increases survival, Cox hazard analysis p=0.008, χ^2^=8.4, n > 590 per genotype. (B) RU does not affect survival of *elav-*GSGal4/+, yet survival is extended in *elav-*GS>*dilp1* without RU because *dilp1* is somewhat over-expressed (leaky) even in the absence of RU by quantitative RT-PCR (Table S2, Supporting Information,). (C) Systemic IIS is not activated by *dilp1* transgene expression driven by *dilp2*-GAL4 in *dilp1; dilp2* null background measured by FOXO and JH response gene mRNA, n=5-6 per genotype, ~15 flies per sample. (D) *Dilp1* transgene expression driven by *dilp2*-GAL4 in *dilp1; dilp2* null background does not affect body size, n=2-3 per genotype, 5-20 flies per replicate. (E) *Dilp1* transgene expression by *dilp2*-GAL4 in *dilp1; dilp2* null background does not affect Akt or ERK phosphorylation (thorax); quantified in (F) pAkt/Akt density, n=6-7 per genotype, 5 flies per sample and (G) pERK/ERK density, n=6-7per genotype, 5 flies per sample. (H) *Dilp3* mRNA*,* but not *dilp5* or *dilp6*, increased by *dilp1* transgene expression driven by *dilp2*-GAL4 in *dilp1; dilp2* null background, n=6-7 per genotype, ~15 flies per sample.

**Fig S6. UAS-*dilp1* expression in a wildtype background extends lifespan but does not inhibit insulin/IGF signaling.**

(A-B) *Dilp1* transgene expression driven by *dilp2*-GAL4 in an otherwise wildtype background extends lifespan in two separate trails: (A) Cox hazard, p<0.0001, χ^2^=92.4, n=328-344 per genotype; (B) Cox hazard p<0.0001, χ^2^=120, n=552-601 per genotype (C-D) *Dilp1* transgene expression driven by *dilp2*-GAL4 does not significantly alter IIS measured by pAkt and pERK: Representative western blots shown (C); (D) Quantification of replicates for pAkt/Akt (left) and pERK/ERK (right), n=4 per genotype, 5 whole flies per sample. (E) In an otherwise wildtype background, *dilp1* transgene driven by *dilp2*-GAL4 decreases *InR* gene expression and increases *Kr-h1* gene expression, but does not alter *Akt* or *4eBP* expression. *p<0.05, n=8 samples per genotype, ~15 whole flies per sample. (F) In an otherwise wildtype background, *dilp1* transgene expression driven by *dilp2*-GAL4 does not alter expression of *dilp3*, *dilp5* or *dilp6,* n=4 samples per genotype.

**References**

Grönke, S., Clarke, D. F., Broughton, S., Andrews, T. D., & Partridge, L. (2010). Molecular evolution and functional characterization of Drosophila insulin-like peptides. *PLoS Genet, 6*(2), e1000857. doi: 10.1371/journal.pgen.1000857

Staber, C. J., Gell, S., Jepson, J. E., & Reenan, R. A. (2011). Perturbing A-to-I RNA Editing Using Genetics and Homologous Recombination. *Methods Mol Bio, 718*, 41-73. doi: doi: 10.1007/978-1-61779-018-8_3
